# Association of patient factors and health-related quality of life with weight changes during chemotherapy for colorectal cancer: secondary analysis of the SCOT trial

**DOI:** 10.1007/s00520-026-10441-3

**Published:** 2026-02-19

**Authors:** T. S. Varghese, S. Faithfull, A. Frampton, A. Harkin, T. Iveson, C. Kelly, M. E. Phillips, M. P. Saunders, A. Lemanska

**Affiliations:** 1 Faculty of Health and Medical Sciences, University of Surrey, Guildford, UK; 2 Trinity St. James’s Cancer Institute, Trinity College Dublin, Dublin, Ireland; 3 School of Cancer and Pharmaceutical Sciences, King’s College London, London, UK; 4 Royal Surrey NHS Foundation Trust, Guildford, UK; 5 Glasgow Oncology Clinical Trials Unit, University of Glasgow, Glasgow, UK; 6 Department of Medical Oncology, University of Southampton, Southampton, UK; 7 The Christie, Manchester, UK; 8 Imperial Clinical Trials Unit, Imperial College London, London, UK

**Keywords:** Chemotherapy, Oxaliplatin, Colorectal cancer, Weight loss, Weight gain, Weight changes

## Abstract

**Purpose:**

To investigate patterns of weight change during chemotherapy for colorectal cancer and analyse their associations with patient demographic, clinical factors, and health-related quality of life (HRQoL).

**Methods:**

We performed secondary analysis of data from 2,871 participants of the SCOT randomised controlled trial. SCOT compared 3 months to 6 months of adjuvant oxaliplatin-containing chemotherapy in people with colorectal cancer. HRQoL was measured with EQ-5D-3L. Mean changes in weight over time were plotted with 95% confidence intervals (CIs) and analysed stratified by sex, chemotherapy regimen and duration. Associations with patient characteristics were evaluated using multivariable linear regression.

**Results:**

The median age of participants was 65 (interquartile range was 59 to 70), 38.7% were female and 95.8% of participants were white. Male participants gained on average 1.2 kg (95% CI 1.0 to 1.4), and females gained 0.8 kg (95% CI 0.6 to 1.1). By the end of treatment, 161 (6.1%) participants shifted from body mass index (BMI) < 25 kg/m^2^ (healthy range) to ≥ 25 kg/m^2^ (overweight and obese), and 73 (2.7%) from BMI ≥ 25 to BMI < 25 kg/m^2^, *p* < 0.001. Furthermore, 210 (7.3%) participants experienced weight loss of more than 5% of their body weight, while 560 (19.5%) gained more than 5%. Change in body weight was associated with age (per 10-year increase -1.9, 95% CI -2.4 to -1.4), male sex (1.6, 95% CI 0.7 to 2.5), and the HRQoL health index (0.6, 95% CI 0.2 to 0.9), all *p* < 0.001.

**Conclusions:**

Younger patients, males, and those with higher HRQoL experienced greater weight gain during chemotherapy for colorectal cancer, emphasising the need to monitor and manage body weight to prevent increase in obesity during curative treatment.

## Highlights

• More people gained weight than lost weight during treatment with females gaining on average 0.8 kg and males 1.2 kg.

• Nearly 20% of participants experienced a weight gain of more than 5% of their baseline body weight and 7.3% experienced a weight loss of more than 5%.

• Male participants were likely to gain more weight during treatment than females.

• The observed association between weight change and health-related quality of life highlighted the potential of dietary interventions during chemotherapy to improve overall health.

## Introduction

Body weight is typically monitored at every cycle in patients undergoing chemotherapy for cancer. Weight changes during chemotherapy may adversely affect long-term post-treatment health and quality of life (QoL), including overall well-being and health-related quality of life (HRQoL). Mechanisms for weight gain during chemotherapy include physical inactivity and metabolic changes due to cancer treatment. Therefore, weight gain is usually associated with negative body composition changes which can cause chronic inflammation, stimulate cancer growth, and metabolic changes. This can significantly increase the risk of cardiovascular disease by contributing to high blood pressure, insulin resistance, type 2 diabetes and dyslipidaemia [1–3]. Being overweight or obese is associated with an increased risk of many cancers [4, 5] and reduced overall survival [6, 7], including in colorectal cancer [4–11]. A study by Sung et al. (2025) examined cancer registry data across 50 countries and documented rising incidence especially among younger adults [5]. Recent reviews by Lei et al. (2021) [8], Mandic et al. (2023), a meta-analysis of cohort studies [9], and Nguyen and Shanmugan (2024) [10], supported the evidence of an increased risk of colorectal cancer, indicating that obese individuals have a 19%—41% higher risk compared with those of normal weight.

Colorectal cancer is the third most commonly diagnosed cancer worldwide and the second leading cause of cancer-related deaths [12]. The increase in early onset among younger populations observed in many countries has been linked to diet, physical inactivity, obesity, and metabolic syndrome [4, 5, 10]. The shift away from traditional diets rich in fibre and low in fat toward those high in sugar, red meat, and ultra-processed foods has been suggested as a contributing factor, with more research needed to investigate colorectal cancer aetiology and epidemiology. Population studies have identified obesity in Western countries to be associated with a 30–70% higher risk of colorectal cancer in men, although the association appears less clear in women [9]. The impact of being overweight or obese during treatment is less well understood, as studies have reported significant differences in cancer-specific outcomes, possibly due to variations in the timing and measures used to assess weight [7]. In a meta-analysis of 45 studies of colorectal cancer survivors, individuals with obesity had a significantly increased risk of overall and cancer-specific mortality compared with those with normal body mass index (BMI) [11].

Traditionally, weight management in cancer has focused around preventing weight loss [13], and survivorship benefits were associated with weight gain [14, 15]. However, this is usually more relevant in the advanced stages of colorectal cancer, where substantial weight loss is a clinically relevant concern for treatment tolerance and survival outcomes, contributing to frailty and cachexia [11]. Avoiding weight gain is a recommendation for cancer survivors to reduce future risk from cancer and reduce treatment adverse effects but also to reduce the risk factors for cancer recurrence [16, 17]. This highlights the need for physical assessments and interventions to maintain healthy weight, muscle mass and function in people treated for colorectal cancer with curative intent [18, 19].

While an increasing number of people survive cancer, a growing population of people faces a range of complex survivorship issues, QoL, physical, psychological, and social challenges [20]. The global prevalence of obesity also continues to rise, and this trend poses significant challenges for cancer incidence and survivorship. Addressing and preventing weight gain is essential to reduce the risk of long-term health complications, and to support overall recovery and QoL. The aim of this study was to investigate weight changes during chemotherapy in people with colorectal cancer, and investigate the association of patient demographics, clinical factors, and HRQoL with weight changes. By understanding weight changes that affect people undergoing cancer treatment, targeted interventions can be developed to prevent unhealthy weight gain, improve treatment outcomes, and enhance long-term health and QoL of cancer survivors.

## Methods

### Data source

We undertook secondary analyses of data from the Short Course Oncology Therapy (SCOT) randomised controlled trial [21]. SCOT was an international, multicentre (244 centres), clinical trial that tested non-inferiority of 3 months versus 6 months of adjuvant oxaliplatin-containing chemotherapy in 6,088 people with high-risk stage II and III colorectal cancer between March 2008 and November 2013. Participants in the trial were randomised to either 3 months or 6 months of chemotherapy. The trial evaluated the disease-free survival and HRQoL [21]. In this study we used data on 2,871 participants from the chemotherapy-induced peripheral neuropathy (CIPN) sub-study of SCOT [22].

Two chemotherapy regimens were allowed in SCOT, fluorouracil with oxaliplatin (FOLFOX) or capecitabine with oxaliplatin (CAPOX). The regimen was decided by clinicians before randomisation. The dose of oxaliplatin in FOLFOX was 85 mg/m^2^ every two weeks and in CAPOX it was 130 mg/m^2^ every three weeks for the duration of treatment which was decided in randomisation. Therefore, the two SCOT randomisation arms were as follows, the 3 months arm (6 cycles of FOLFOX delivered every 2 weeks or 4 cycles of CAPOX delivered every 3 weeks) and the 6 months arm (12 cycles of FOLFOX delivered every 2 weeks or 8 cycles of CAPOX delivered every 3 weeks).

### Study design

In these secondary analyses of SCOT data, we undertook longitudinal analysis and investigated weight changes from baseline to the completion of chemotherapy. We presented the results separately for males and females and stratified the analyses by chemotherapy duration and regimen.

### Participants and data

The study sample included 2,871 participants from the CIPN sub-study of SCOT [22], which was conducted as per the SCOT design reported in Iveson et al. 2018 [21]. We accessed the information on age, sex, height, and weight at baseline and at every cycle, SCOT randomisation arm (chemotherapy duration), and chemotherapy regimen. As per the SCOT protocol, adults who had undergone curative resection for high-risk stage II or stage III adenocarcinoma of the colon or rectum were enrolled within 11 weeks of surgery and started chemotherapy within 2 weeks of randomisation to their allocated study group. Only participants with a WHO performance status of 0 or 1, adequate organ function, and a life expectancy greater than 5 years (excluding cancer-related disease) were eligible for inclusion in the SCOT trial. In the 3 months arm, the total oxaliplatin dose was 510 mg/m^2^ in the FOLFOX regimen and 520 mg/m^2^ in the CAPOX regimen, and in the 6 months arm it was 1020 mg/m^2^ and 1040 mg/m^2^ respectively. No dose reductions of oxaliplatin were allowed at baseline. The analysis was as per protocol, with no participants excluded due to chemotherapy dose reductions, discontinuation or toxicity. HRQoL was assessed with the EuroQol five-dimensional questionnaire using the three-level (EQ-5D-3L) visual analogue scale in five domains of mobility, self-care, usual activities, pain/discomfort, and anxiety/depression, as well as the EQ-5D health index, derived with the UK value sets [23].

### Outome measures

We analysed two outcome measures. Body weight which was recorded for all participants at every cycle. Based on weight, we also calculated BMI at every cycle using height which was recorded at baseline. We assessed weight changes in kg, BMI units (kg/m^2^), and BMI categories. We categorised participants as normal weight, BMI < 25 kg/m^2^, and overweight and obese, BMI ≥ 25 kg/m^2^. This was due to the small number of participants classified as underweight at baseline (39, 1.4%) and at the end of treatment (35, 1.2%), the underweight category was not analysed separately.

### Statistical analysis

Baseline participant characteristics, age, sex, ethnicity, performance status, disease site (colon or rectum), and chemotherapy regimen were presented using descriptive statistics, means with standard deviations, medians with interquartile ranges, and counts with percentages. Additionally, we reported numbers of participants who experienced weight loss or gain of more than 5% during the treatment period.

Longitudinal trends in body weight in kg were visualised with means and 95% confidence intervals (CIs) from the start of treatment (baseline) to the end, stratifying by sex (male vs female), chemotherapy duration (3 months vs 6 months), and chemotherapy regimen (FOLFOX vs CAPOX). Changes in body weight from the start of treatment to the end were analysed separately in males and females. We reported this in kg, BMI units, and BMI categories. We reported this for the total cohort as well as stratifying by chemotherapy duration and chemotherapy regimen. We used t-test and chi-squared test to compare the data between groups due to chemotherapy duration and regimen. To estimate the statistical significance of changes from the start to the end of treatment we used paired t-test and McNemar's chi-squared test for paired data.

Liner regression was used to investigate the link of weight changes with patient and clinical factors, and the last recorded HRQoL. Age and HRQoL were scaled in units of 10 to aid interpretability of the results. Weight change was expressed as slope obtained by a linear model which took into account dates, so accounted for the exact number of days between cycles for each participant. We then standardised the rate of change between the 3 months and 6 months groups. The slope was normalised based on treatment duration so that it estimated the predicted change over a 6-month period for each participant.

We investigated the relationship of weight change with age, sex, ethnicity, cancer site, performance status, baseline BMI, HRQoL domains of mobility, self-care, usual activities, pain and discomfort, anxiety and depression, as well as health index. The model was adjusted for chemotherapy duration and regimen. We presented univariable and multivariable models with coefficients and their 95% CIs. We assessed multicollinearity among the independent variables using Variance Inflation Factor (VIF) values. VIF values were between 1.0 and 2.2 so below the threshold of 5, indicating no multicollinearity concerns. Therefore, all variables were retained in the multivariable linear regression model.

Data analyses were undertaken according to the intention-to-treat principle. The proportions of missing data were described but missing data were not imputed, and we used complete case analyses. Statistical significance was considered at the cut-off value of *p* < 0.01 to account for multiple statistical testing. The analyses were performed in R version 4.0.2.

### Ethical considerations

The study protocol was approved by the SCOT steering group. Ethical approval (FHMS 23–24 066 EGA) was granted by the University of Surrey Ethics Committee on 18th January 2024.

## Results

### Sample characteristics

The median age of participants was 65 years (interquartile range [IQR] 59 to 70). Most participants were male (1,761, 61.3%) and of White ethnicity (2,694, 95.8%). Baseline characteristics of the study cohort were presented in Table 1. As previously reported, they did not differ across randomisation arms and chemotherapy regimens [21, 22]. As per the SCOT’s design, participants were equally assigned to the 3 months and 6 months arms with the same proportions in each arm receiving FOLFOX (32%) or CAPOX (68%). EQ-5D-3L questionnaires were available for 1,781 (62.0%) participants.

**Table 1:** Baseline demographic and clinical characteristics of the study population (N = 2,871) presented as counts (n) and percentages. Age was summarised with mean and standard deviation (SD) and median with interquartile range (IQR)

Characteristic	Summary statistic	*n*	%
Age (years)			
Mean (SD)	63.5 (9.4)		
Median (IQR)	65 (59 to 70)		
Missing		0	0.0%
Sex			
Female		1,110	38.7%
Male		1,761	61.3%
Missing		0	0.0%
Ethnicity			
White		2,694	95.8%
Other		118	4.2%
Missing		59	2.1%
Performance status score			
0		1,958	68.2%
1		913	31.8%
Missing		0	0.0%
Disease site			
Colon		2,324	80.9%
Rectum		547	19.1%
Missing		0	0.0%
Chemotherapy regimen			
FOLFOX		925	32.2%
CAPOX		1,946	67.8%
Missing		0	0.0%
More than 5% change in body weight			
Lost > 5% body weight		210	7.3%
Females		92	8.3%
Males		118	6.7%
Gained > 5% body weight		560	19.5%
Females		202	18.2%
Males		358	20.3%

### Changes in body weight

By the end of treatment, on average, female participants gained 0.8 kg (95% CI 0.6 to 1.1, *p* < 0.001) and male participants 1.2 kg (95% CI 1.0 to 1.4, *p* < 0.001). At the start of treatment, the average weight for females was 68.7 kg (95% CI 67.8 to 69.5) and for males 83.4 kg (95% CI 82.7 to 84.1), while at the end of treatment it was 69.5 (95% CI 68.6 to 70.4) and 84.6 (95% CI 83.9 to 85.4) for females and males respectively. This was presented in Table 2, for the overall sample and with stratification by chemotherapy duration and regimen.

**Table 2:** Changes from the start of treatment to the end of treatment in body weight in kg, body mass index (BMI) in kg/m^2^, and BMI categories, BMI ≤ 25 or BMI > 25. As appropriate, t-test, paired t-test, chi-squared test, and McNemar's chi-squared test for paired data were used to assess statistical significance. Means were presented with 95% confidence intervals (CI). The analyses were stratified by randomisation arm (3 months vs 6 months), and by treatment regimen (FOLFOX vs CAPOX). Statistical significance is considered at the cut-off value of 0.01

	Total sample, *N* = 2,871	Randomisation arm		*p*-value	Treatment regimen		*p*-value
		3 months arm, *n* = 1,445	6 months arm, *n* = 1,426		FOLFOX, *n* = 925	CAPOX, *n* = 1,946	
Body weight (kg)							
Start of treatment							
Female, mean (95% CIs)	68.7 (67.8 to 69.5)	69.1 (67.9 to 70.4)	68.2 (67.0 to 69.4)	0.285	68.4 (66.9 to 69.9)	68.8 (67.7 to 69.8)	0.681
Male, mean (95% CIs)	83.4 (82.7 to 84.1)	83.0 (82.0 to 84.0)	83.8 (82.8 to 84.8)	0.289	84.0 (82.8 to 85.3)	83.1 (82.3 to 84.0)	0.246
Missing, *n* (%)	34 (1.2%)	17 (1.2%)	17 (1.2%)		11 (1.2%)	23 (1.2%)	
End of treatment							
Female, mean (95% CIs)	69.5 (68.6 to 70.4)	69.0 (67.8 to 70.3)	69.9 (68.6 to 71.3)	0.351	68.9 (67.3 to 70.5)	69.8 (68.6 to 70.9)	0.394
Male, mean (95% CIs)	84.6 (83.9 to 85.4)	83.9 (82.8 to 84.9)	85.4 (84.4 to 86.5)	0.034	85.3 (84.0 to 86.6)	84.3 (83.4 to 85.2)	0.188
Missing, *n* (%)	194 (6.8%)	99 (6.9%)	95 (6.7%)		37 (4.0%)	157 (8.1%)	
Difference between baseline and end							
Female, value (95% CIs)	0.8 (0.6 to 1.1)	−0.1 (−0.4 to 0.2)	1.6 (1.2 to 2.0)		0.6 (0.2 to 1.0)	1.0 (0.7 to 1.3)	
*p*-value (paired t-test)	< 0.001	0.500	< 0.001		0.007	< 0.001	
Male, value (95% CIs)	1.2 (1.0 to 1.4)	0.9 (0.6 to 1.1)	1.6 (1.3 to 1.9)		1.4 (1.0 to 1.7)	1.2 (0.9 to 1.4)	
*p*-value (paired t-test)	< 0.001	< 0.001	< 0.001		< 0.001	< 0.001	
BMI (kg/m^2^)							
Start of treatment							
Female, mean (95% CIs)	26.4 (26.1 to 26.7)	26.6 (26.1 to 27.1)	26.2 (25.8 to 26.7)	0.257	26.3 (25.7 to 26.8)	26.5 (26.1 to 26.9)	0.489
Male, mean (95% CIs)	27.3 (27.1 to 27.5)	27.2 (26.8 to 27.5)	27.4 (27.1 to 27.7)	0.262	27.4 (27.0 to 27.8)	27.2 (27.0 to 27.5)	0.522
Missing, *n* (%)	43 (1.5%)	23 (1.6%)	20 (1.4%)		12 (1.3%)	31 (1.6%)	
End of treatment							
Female, mean (95% CIs)	26.8 (26.1 to 26.7)	26.6 (26.1 to 27.1)	26.9 (26.4 to 27.4)	0.305	26.5 (25.9 to 27.1)	26.9 (26.4 to 27.3)	0.329
Male, mean (95% CIs)	27.7 (27.1 to 27.5)	27.4 (27.1 to 27.8)	28.0 (27.7 to 28.3)	0.027	27.8 (27.5 to 28.2)	27.6 (27.3 to 27.9)	0.388
Missing, *n* (%)	199 (6.9%)	103 (7.1%)	96 (6.7%)		37 (4.0%)	162 (8.3%)	
Difference between baseline and end							
Female, value (95% CIs)	0.3 (0.2 to 0.4)	0.0 (−0.1 to 0.1)	0.6 (0.5 to 0.8)		0.2 (0.1 to 0.4)	0.4 (0.3 to 0.5)	
*p*-value (paired t-test)	< 0.001	0.571	< 0.001		0.008	< 0.001	
Male, value (95% CIs)	0.4 (0.3 to 0.5)	0.3 (0.2 to 0.4)	0.5 (0.4 to 0.6)		0.4 (0.3 to 0.6)	0.4 (0.3 to 0.5)	
*p*-value (paired t-test)	< 0.001	< 0.001	< 0.001		< 0.001	< 0.001	
BMI (kg/m^2^) categorised							
Start of treatment							
< 25, *n* (%)	1,048 (37.1%)	523 (36.8%)	525 (37.3%)	0.787	339 (37.1%)	709 (37.0%)	0.989
≥ 25, *n* (%)	1,780 (62.9%)	899 (63.2%)	881 (62.7%)		574 (62.9%)	1,206 (63.0%)	
Missing, *n* (%)	43 (1.5%)	23 (1.6%)	20 (1.4%)		12 (1.3%)	31 (1.6%)	
End of treatment							
< 25, *n* (%)	896 (33.5%)	462 (34.4%)	434 (32.6%)	0.346	292 (32.9%)	604 (33.9%)	0.646
≥ 25, *n* (%)	1,776 (66.5%)	880 (65.6%)	896 (67.3%)		596 (67.1%)	1,180 (66.1%)	
Missing, *n* (%)	199 (6.9%)	103 (7.1%)	96 (6.7%)		37 (4.0%)	162 (8.3%)	
Difference between baseline and end							
No change, *n* (%)	2,426 (91.2%)	1,235 (92.3%)	1,191 (90.1%)		801 (90.5%)	1,625 (91.5%)	
< 25 to ≥ 25, *n* (%)	161 (6.1%)	67 (5.0%)	94 (7.1%)		60 (6.8%)	101 (5.7%)	
≥ 25 to < 25, *n* (%)	73 (2.7%)	36 (2.7%)	37 (2.8%)		24 (2.7%)	49 (2.8%)	
*p*-value (McNemar's test)	< 0.001	0.003	< 0.001		< 0.001	< 0.001	

**Fig. 1:**
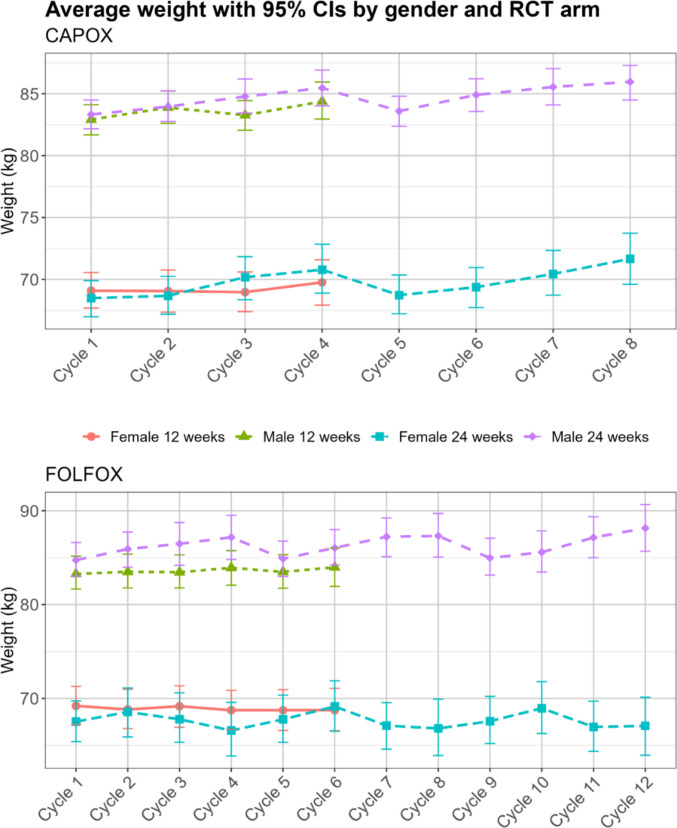
Trends in mean body weights with 95% confidence intervals (CIs) stratified by sex, chemotherapy regimen (CAPOX top and FOLFOX bottom plot), and chemotherapy duration (3 months vs 6 months). The CAPOX regimen had either 4 or 8 cycles delivered every 3 weeks. The FOLFOX regimen had either 6 or 12 cycles delivered every 2 weeks

Longitudinal trends in body weight (Fig. 1) showed fluctuations during treatment, with greater weight gain in the 6 months arm and among male participants, who gained nearly 2 kg in the FOLFOX regimen. In the 3 months arm, female weight did not change (*p* = 0.500) while for males it increased by 0.9 kg (95% CI 0.6 to 1.1, *p* < 0.001, Table 2). In the 6 months arm, females and males gained 1.6 kg (*p* < 0.001). BMI for females increased by 0.3 kg/m^2^ (95% CI 0.2 to 0.4, *p* < 0.001), from 26.4 kg/m^2^ (95% CI 26.1 to 26.7) at the start of treatment to 26.8 kg/m^2^ (95% CI 26.1 to 26.7) at the end. For males it increased by 0.4 kg/m^2^ (95% CI 0.3 to 0.5, *p* < 0.001) from 27.3 kg/m^2^ (95% CI 27.1 to 27.5) to 27.7 kg/m^2^ (95% CI 27.1 to 27.5). At the start of treatment 1,048 (37.1%) participants were of normal weight, but161 (6.1%) participants shifted to overweight or obese by the end of treatment, while 73 (2.7%) shifted from overweight or obese to normal BMI.

### Association of patient and clinical factors with weight changes

The multivariable regression indicated associations of weight change with age, sex, and the EQ-5D health index (Table 3). Age was negatively associated with weight change. For every 10-year increase in age, the rate of weight gain decreased by 1.9 kg (95% CI 1.4 to 2.4, *p* < 0.001). Health index was positively associated with weight gain, with each 10-unit increase in the health index corresponding to a 0.6 kg increase in weight (95% CI 0.2 to 0.9, *p* < 0.001). Additionally, male sex was associated with greater weight gain compared to female sex, with males experiencing, on average, a 1.6 kg higher increase in weight (95% CI 0.7 to 2.5, *p* < 0.001). After adjusting for other covariates, ethnicity, cancer site, performance status, baseline BMI, and HRQoL domains (mobility, self-care, usual activities, pain/discomfort, and anxiety/depression) were not associated with weight change.

**Table 3:** Results of linear regression to investigate the link between weight changes during chemotherapy and patient demographics, clinical factors, and health-related quality of life (HRQoL) measured with the EuroQol five-dimensional 3-level (EQ-5D-3L) questionnaire in domains of mobility, self-care, usual activities, pain/discomfort, anxiety/depression, as well as the health index. Weight change was expressed as a slope of a linear model which took number of days between cycles into account. We used univariable and multivariable models and presented the results as regression coefficients, with 95% confidence intervals (CI) and p-values. Statistical significance was considered at the cut-off value of 0.01

	Univariable models			Multivariable model		
6 months	Coefficient	95% CI	*p*-value	Coefficient	95% CI	*p*-value
Age (10 years)	−2.0	−2.3 to −1.6	< 0.001	−1.9	−2.4 to −1.4	< 0.001
Sex (male)	1.3	0.6 to 1.9	< 0.001	1.6	0.7 to 2.6	< 0.001
Ethnicity (white)	0.2	−1.5 to 1.9	0.840	1.4	−0.9 to 3.8	0.223
Cancer site (rectum)	0.0	−0.8 to 0.9	0.940	0.2	−1.0 to 1.3	0.754
Performance status (1)	−0.9	−1.6 to −0.2	0.014	−0.1	−1.0 to 0.8	0.813
Baseline BMI	−0.1	−0.2 to 0.0	0.012	−0.1	−0.2 to 0.0	0.245
Regimen (CAPOX)	0.2	−0.5 to 0.9	0.605	0.2	−0.7 to 1.2	0.667
Randomisation arm (6 months)	0.2	−0.4 to 0.9	0.471	−0.2	−1.1 to 0.7	0.602
Mobility	−0.5	−1.5 to 0.5	0.316	1.0	−0.4 to 2.4	0.145
Self-care	0.4	−1.0 to 1.8	0.581	1.0	−0.8 to 2.8	0.264
Usual activities	−0.1	−1.0 to 0.8	0.797	0.3	−1.1 to 1.6	0.687
Pain/discomfort	−0.1	−0.9 to 0.7	0.785	0.3	−0.8 to 1.3	0.637
Anxiety/depression	0.4	−0.4 to 1.3	0.312	0.2	−0.9 to 1.3	0.671
Health index	0.4	0.1 to 0.6	0.004	0.6	0.2 to 0.9	< 0.001

### Missing data

Ethnicity was missing for 59 (2.1%) study participants. Baseline weight was missing for 34 (1.2%) participants, and height was missing for further 9 participants, so BMI was missing for 43 (1.5%). No missing data were reported for other patient and clinical factors at baseline. Out of the 1,781 participants who provided EQ-5D-3L questionnaires, four did not have data after the end of treatment, with additional 18, 24, 25, 25, 21, and 124 participants with intermittent missing data for mobility, self-care, usual activities, pain and discomfort, anxiety and depression, and health index.

## Discussion

### Summary of findings

In this study, by the end of treatment, 6.1% of participants shifted from normal weight BMI category to overweight or obese, while only 2.7% shifted from overweight or obese to normal weight. Nearly 20% of participants experienced weight gain of more than 5% of their baseline body weight, and 7.3% experienced weight loss of more than 5%. In addition, males had a 1.6-unit greater rate of change in body weight over time compared to females. Lee et al. (2020) found similar rates among 2,455 participants, over 26% experienced weight gain of over 5 kg and 7.3% experienced weight loss over 5 kg, with weight gain more frequently observed in males [24]. Our findings demonstrate that younger age, male sex, and higher EQ-5D health index were associated with greater weight gain during the six-month treatment period. These results align with previous studies showing that men are more likely than women to regain or maintain weight following colorectal cancer treatment [25, 26]. In addition, older patients were more likely to experience weight loss during treatment, possibly linked to age-related changes in metabolism, comorbidities, and reduced physiological functioning [27].

Our findings highlighted the bidirectional nature of weight changes during chemotherapy, with weight gain and weight loss observed in notable proportions of patients, contributing to the growing body of evidence that weight changes, are clinically important, and require monitoring and personalised approaches to optimise outcomes in colorectal cancer. Early interventions, including referral to a dietitian during chemotherapy, may help mitigate weight changes and reduce associated risks [28, 29]. The World Cancer Research Fund and American Institute for Cancer Research published dietary and nutritional guidelines for cancer survivors, however, specific recommendations for colorectal cancer remain limited [30].

### Discussion in the context

Most of the existing evidence on the role of obesity in cancer survivorship comes from studies of breast and prostate cancer, while research in colorectal cancer has primarily focused on BMI prior to diagnosis and treatment, with conflicting findings related to treatment-related adverse effects and survival. In our study, 63% of patients were overweight or obese at the start of treatment, increasing to 67% by the end of treatment. Kenkhuis et al. (2023), in a study of 459 colorectal cancer survivors, reported similar rates, with 44% overweight and 31% obese at diagnosis [31]. They also assessed body composition, including adiposity, from diagnosis through treatment and up to 24 months post-treatment. Consistent with our findings, they observed that patients with higher BMI and greater adiposity had better HRQoL.

While research has primarily focused on investigating weight loss as a marker of poor prognosis in palliative patients [32], increasing number of studies point to excessive weight gain as a concern in curative treatment [14, 33–38]. A meta-analysis of 16 cohort studies (58,917 patients) by Lee et al. (2015) demonstrated that being obese prior to diagnosis was associated with increased colorectal cancer-specific mortality and all-cause mortality, whereas being obese after diagnosis was associated with increased all-cause mortality [38]. A meta-analysis of 16 studies involving 55,391 patients by Li et al. (2022) concluded that higher BMI was associated with more favourable outcomes, whereas both underweight and morbidly obese patients had worse overall survival compared with those of normal weight [7]. This highlights the variability and limitations in outcomes across studies, indicating a need for further research. BMI can be a poor indicator of body composition, and more detailed assessments may provide a better understanding of changes related to cancer therapy. Chemotherapy dosing is commonly based on body size parameters such as weight, height, body surface area, or dose banding, which can lead to dose capping and result in unreliable dosing strategies, with a risk of underdosing or overdosing and increased toxicity. Stocker et al. (2018), in a study of adjuvant chemotherapy for colorectal cancer, found that dose reductions in obese patients negatively affected disease-free survival [39]. Although poorer outcomes among overweight and obese patients are multifactorial, guidelines on personalised dosing and monitoring in obesity are emerging [40], highlighting the need for continued research in this area.

Many studies examining the effects of weight loss have focused on women with breast cancer. A systematic review and meta-analyses of 17 reviews reported that weight loss interventions can reduce treatment-related adverse effects and lead to beneficial outcomes in this population [41]. However, less is known about the impact of such interventions on outcomes and recurrence in colorectal cancer. Patients with colorectal cancer face specific challenges and concerns and in relation to diet and managing bowel changes after surgery and chemotherapy [28], and therefore, interventions developed for other cancer populations may not be directly generalisable. A synthesised guideline derived from international recommendations on diet and physical activity for colorectal cancer survivors highlighted that existing guidance specific to colorectal cancer is limited and primarily focused on improving general health [42].

### Strengths and limitations

This study benefitted from rigorous data collection within the RCT setting, with body weights and precise dates recorded at every treatment cycle, allowing the exact number of days between weight measurements to be accounted for in the analysis. The homogeneity of the cohort was a strength. All participants had stage II or III colorectal cancer, underwent surgery, and received adjuvant oxaliplatin-fluoropyrimidine combination therapy. This enhanced the internal validity in assessing weight changes. However, the randomised variation in chemotherapy duration, ranging from three to six months, posed a challenge for a direct comparison of weight changes across patients. Detailed patient-level information on cancer stage, surgical approach, time between surgery and the start of chemotherapy, and the presence of a stoma was not available and therefore could not be included in the analysis as potential confounding factors, which may have influenced energy expenditure and food intake. The lack of long-term post-treatment weight measurements limited the analysis of weight changes to six months. Additionally, as the study focused solely on the treatment period, we did not have access to recovery, recurrence and mortality data so did not investigate the link of weight changes with survival. The absence of detailed lifestyle data, including information on dietary intake and physical activity, restricted the ability to account for these important confounders. Finally, the findings may have limited generalisability to broader clinical populations outside of RCTs.

## Conclusions

In conclusion, our study demonstrated that age, sex, and HRQoL were associated with weight change during curative treatment for colorectal cancer. Younger patients, males, and those with higher HRQoL were more likely to experience greater weight gain over the treatment period. These findings highlighted the need for supportive care strategies that include lifestyle and nutrition, and account for demographic and psychosocial factors in colorectal cancer management.

## Data Availability

Data were provided by the Glasgow Oncology Clinical Trials Unit with the approval of the SCOT steering group.
